# Supplementary material for: Housing temperature influences exercise training adaptations in mice
Source: Nat Commun. 2020 Mar 25;11:1560. doi: 10.1038/s41467-020-15311-y (PMC7096511; doi:10.1038/s41467-020-15311-y)
Supplement: Supplementary file 1 — Supplementary Information [file 41467_2020_15311_MOESM1_ESM.pdf]

Housing temperature influences exercise training adaptations in mice

Supplementary information

Raun et al.

Supplementary Figure 1

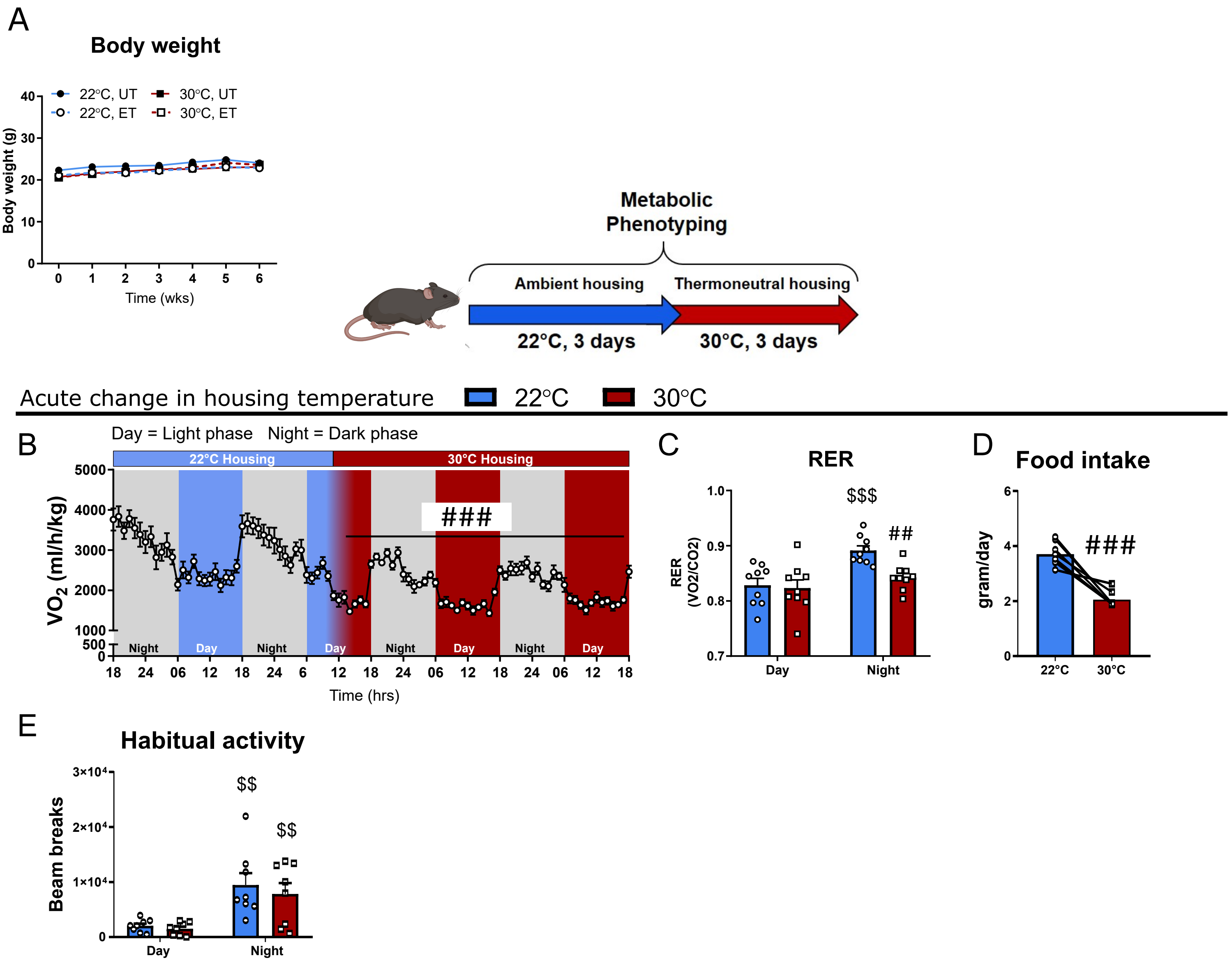

Supplementary figure 1:

(A) The effect of housing temperature and exercise training (ET) at 22°C and 30°C on bodyweight (gram). n=8-10.

(B-E): The effect of acute change of temperature from 22°C to 30°C on oxygen uptake (VO_2), respiratory exchange ratio (RER), food intake, and ambulant activity (2 consecutive days). n=9. Effect of time; $$ p<0.01, $$$ p<0.001. Effect of temperature; ## p<0.01, ### p<0.001.

Data are presented as mean ± SEM incl. individual values where applicable. The “n=x” defines the number of biologically independent animals used for the analyses.

Supplementary
Figure 2

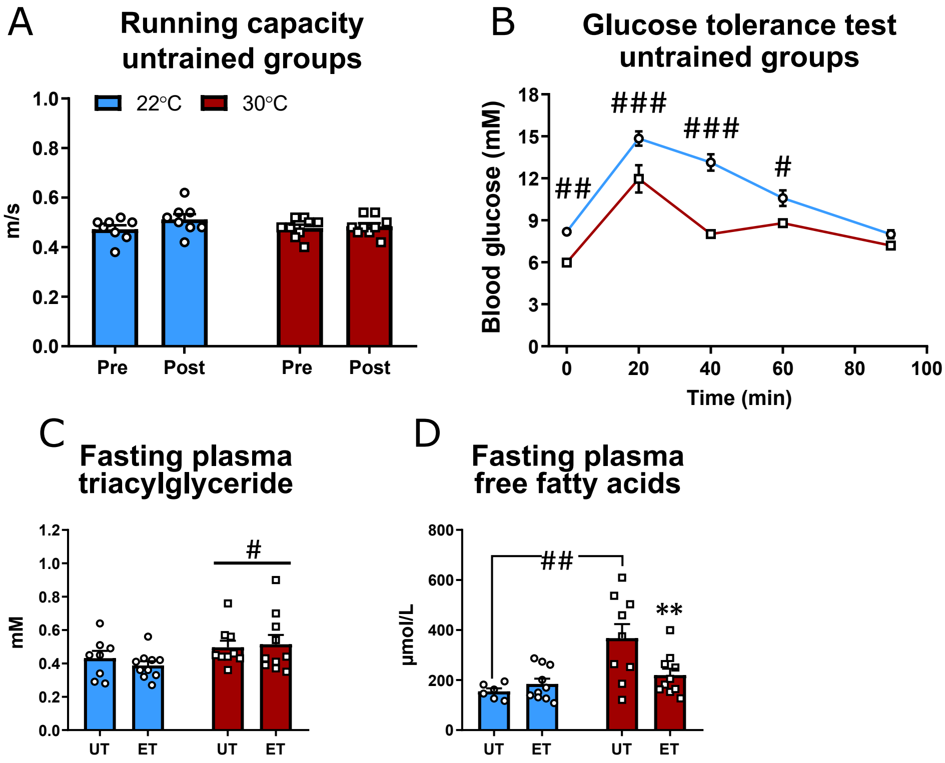

Supplementary figure 2:

(A): Exercise capacity before (Pre) and after (Post) the intervention in the control mice. n=8-10.

(B) The effect of housing temperature on glucose tolerance in control mice. n=8-9. Effect of temperature; # $p<0.05$, ## $p<0.01$, ### $p<0.001$.

(C-D) Effect of housing temperature and exercise training (ET) at 22°C and 30°C on fasting plasma triglycerides (C) and fasting free fatty acids (D). n=8-10. Effect of ET within temperature; * $p<0.05$. Effect of temperature; # $p<0.05$, ## $p<0.01$.

Data are presented as mean ± SEM incl. individual values. The "n=x" defines the number of biologically independent animals used for the analyses.

Supplementary Figure 3

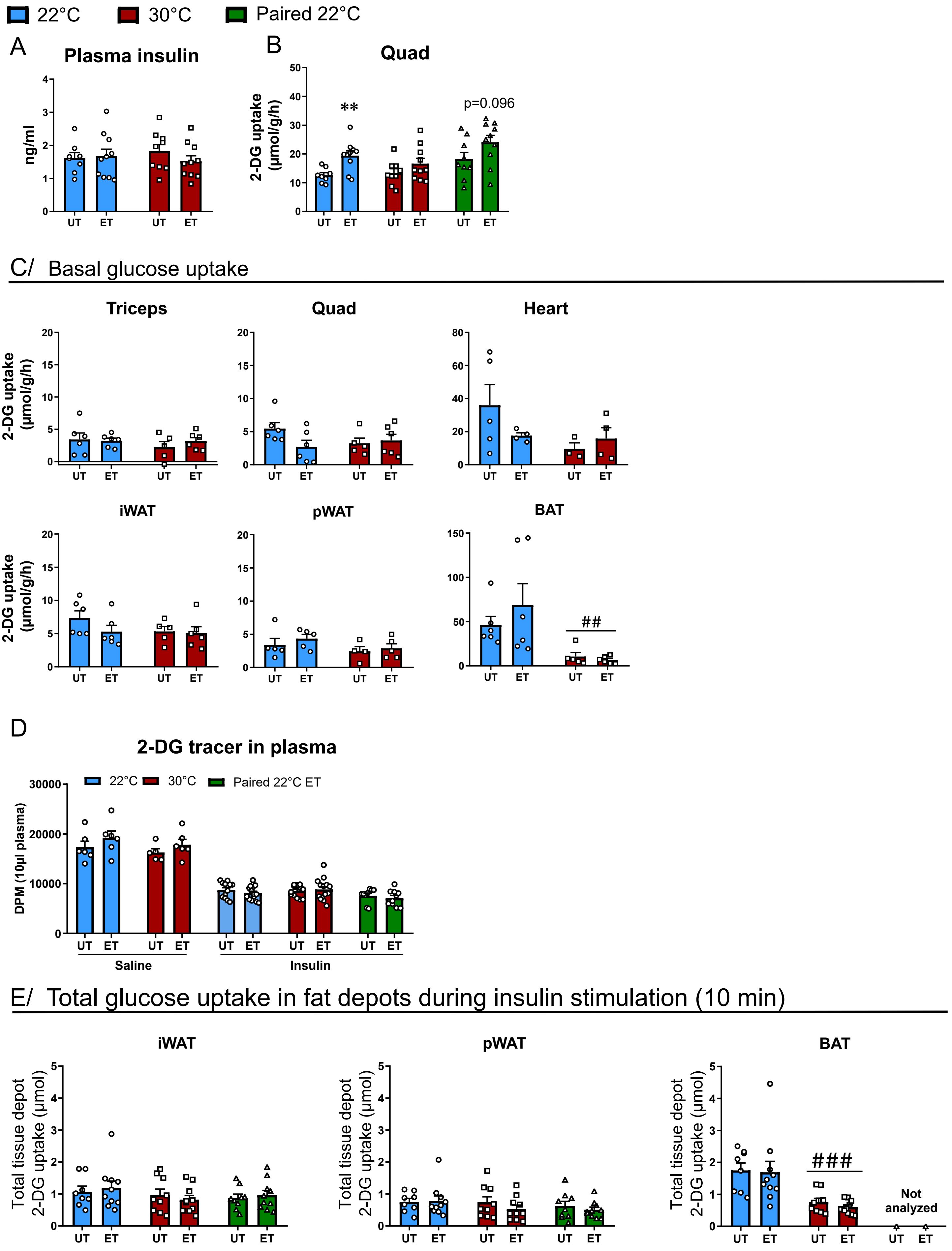

Supplementary figure 3:

(A) Plasma insulin after retro-orbital insulin injection (at min 10). n=8-10.

(B) Effect of exercise training (ET) on insulin-stimulated glucose uptake at 22°C, 30°C, and paired 22°C in skeletal muscle (m. quadriceps). n=8-10. Effect of ET within temperature; * $p<0.05$.

(C) Basal glucose uptake in all experimental groups. n=6. Effect of temperature; ## $p<0.01$.

(D) 2-DG plasma tracer counts of all experimental groups

(E) Effect of ET on total adipose tissue depot glucose uptake during 10min insulin stimulation in 22°C, 30°C, and paired 22°C in iWAT, pWAT, and BAT. n=8-10. Effect of temperature as indicated with lines; ### $p<0.001$.

Data are presented as mean ± SEM incl. individual values. The “n=x” defines the number of biologically independent animals used for the analyses.

Supplementary Figure 4

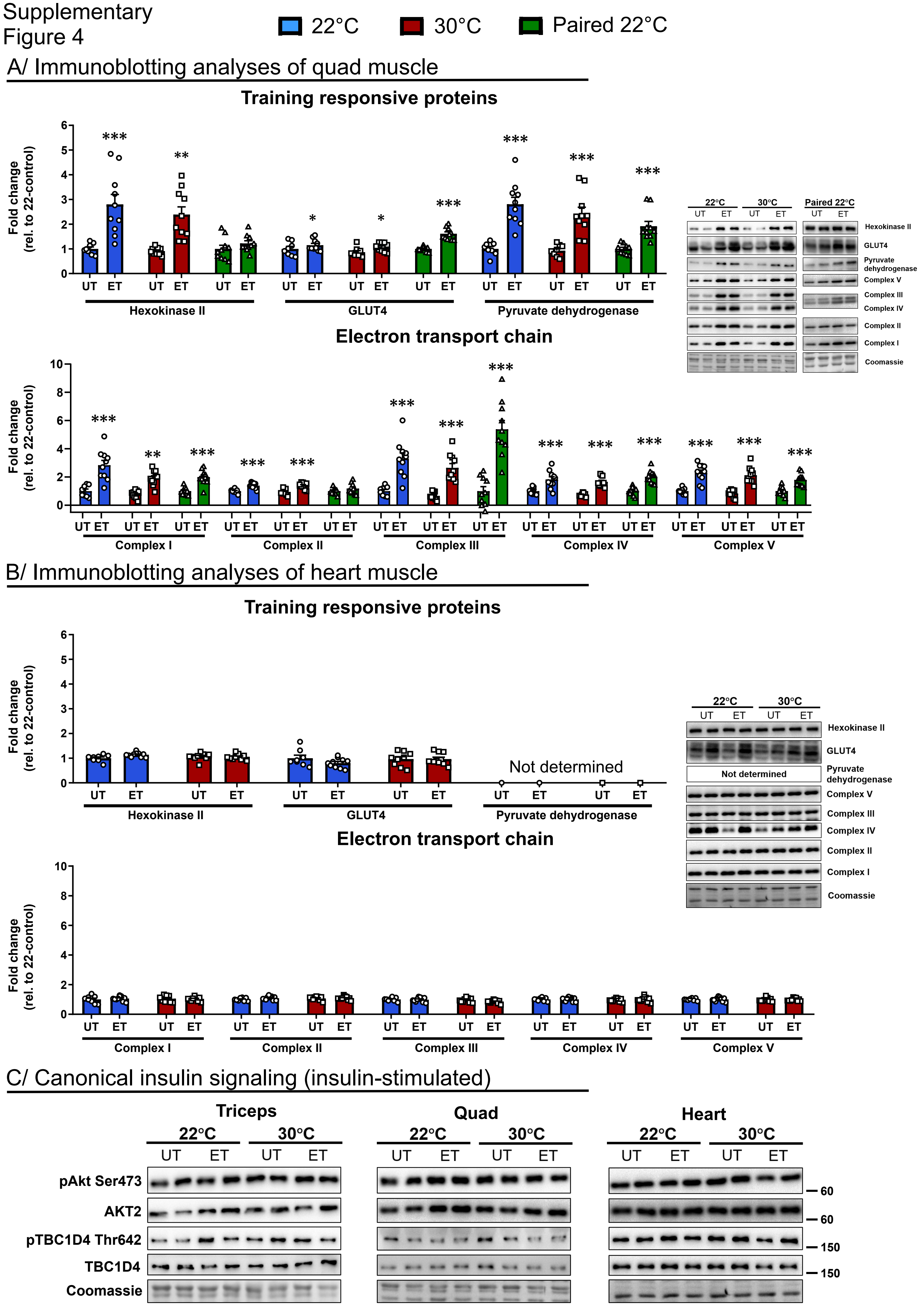

Supplementary figure 4:

(A) Effect of exercise training (ET) on training responsive proteins and subunits of the electron transport chain of the mitochondrion in quadriceps muscle in 22°C, 30°C, and paired 22°C. n=8-10. Effect of ET within temperature; * $p<0.05$, ** $p<0.01$, *** $p<0.001$.

(B) Effect of ET on training responsive proteins and subunits of the electron transport chain of the mitochondrion in heart muscle in 22°C and 30°C. n=8-10.

(C) Representative blots of canonical insulin signaling in all muscles investigated.

Data are presented as mean ± SEM incl. individual values. The "n=x" defines the number of biologically independent animals used for the analyses.

Supplementary
Figure 5

Genes involved in thermogenesis (qPCR)

22°C   30°C

A/ iWAT

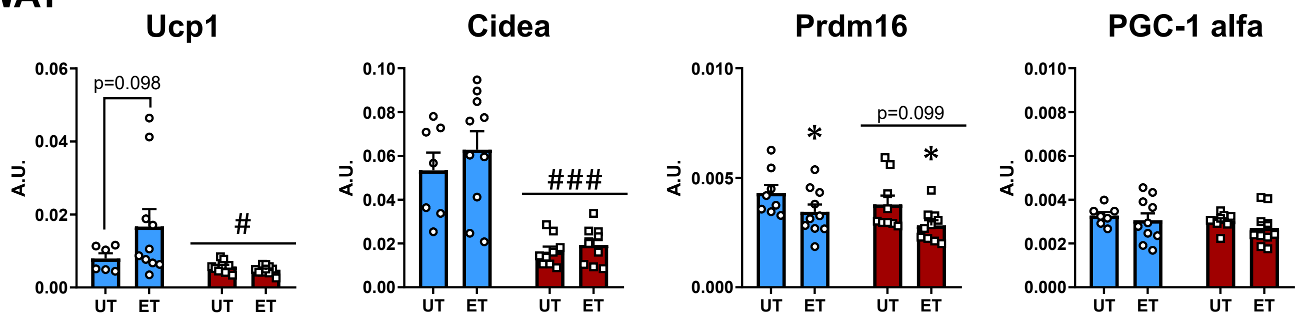

B/ BAT

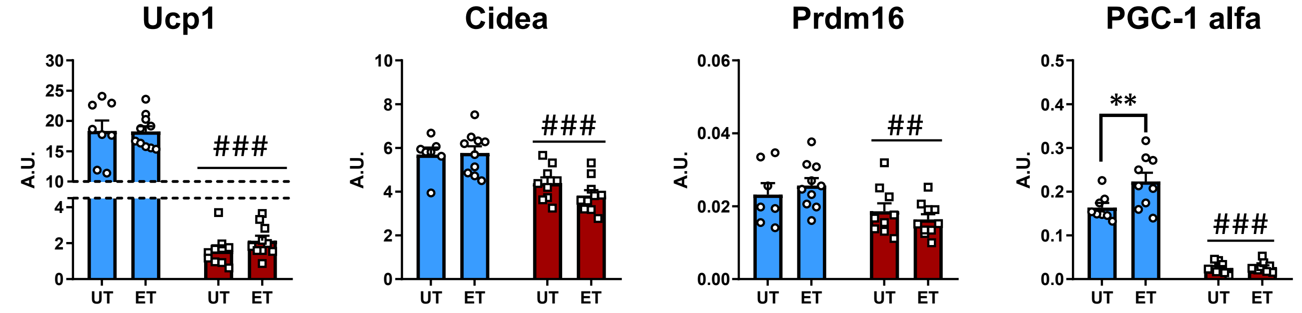

C/ Uncontrained Bray-Curtis PCoA plot displaying the time effect and Adonis tests quantifying variance between the gut microbiome (related to figure 6A)

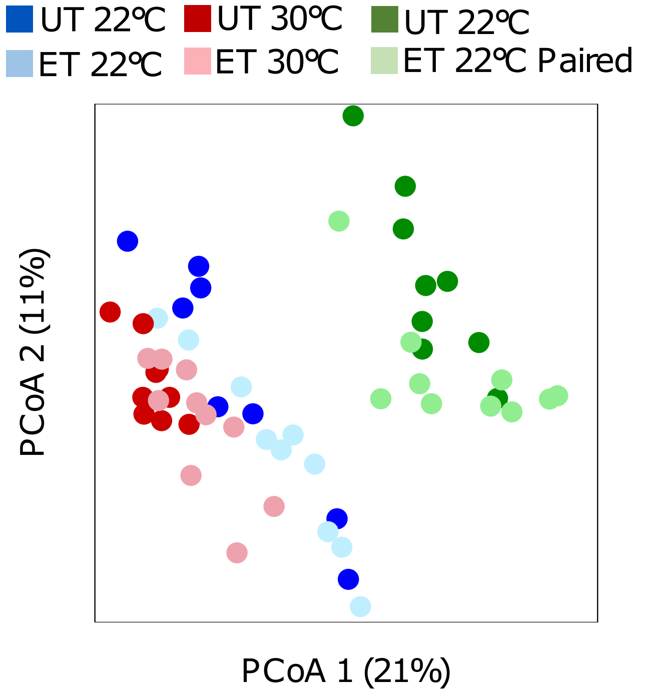

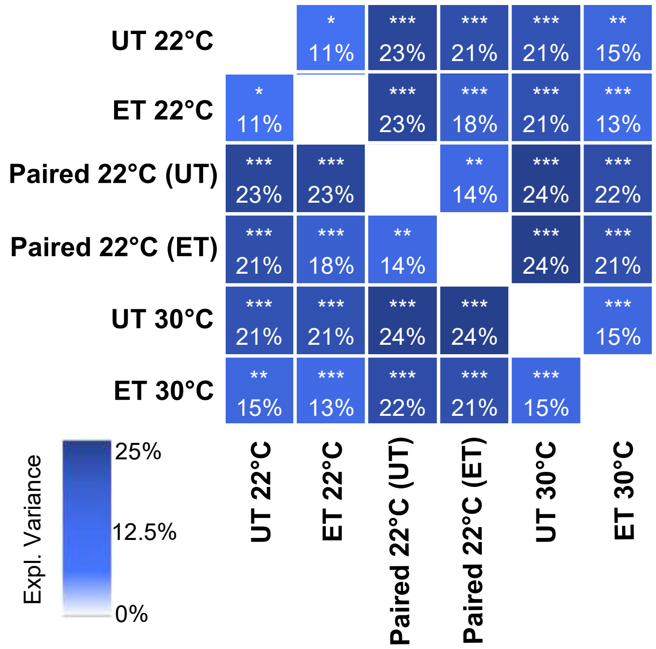

Supplementary figure 5:

(A-B) Thermo-regulatory genes in iWAT (A) and BAT (B) depots. n=7-10. Effect of ET within temperature; * $p<0.05$. Effect of temperature within UT or ET groups; ## $p<0.01$, ### $p<0.001$.

(C) Left: unconstrained Bray-Curtis PCoA plot displaying the time effect of the experimental units. Right: Adonis tests quantifying (significant) variance explained between GM according to experimental groups treatments. Adonis test was based on Bray-Curtis zOTU distance matrix. *$P<0.05$, **$P<0.01$, ***$P<0.001$.

Data are presented as mean ± SEM incl. individual values. The "n=x" defines the number of biologically independent animals used for the analyses.

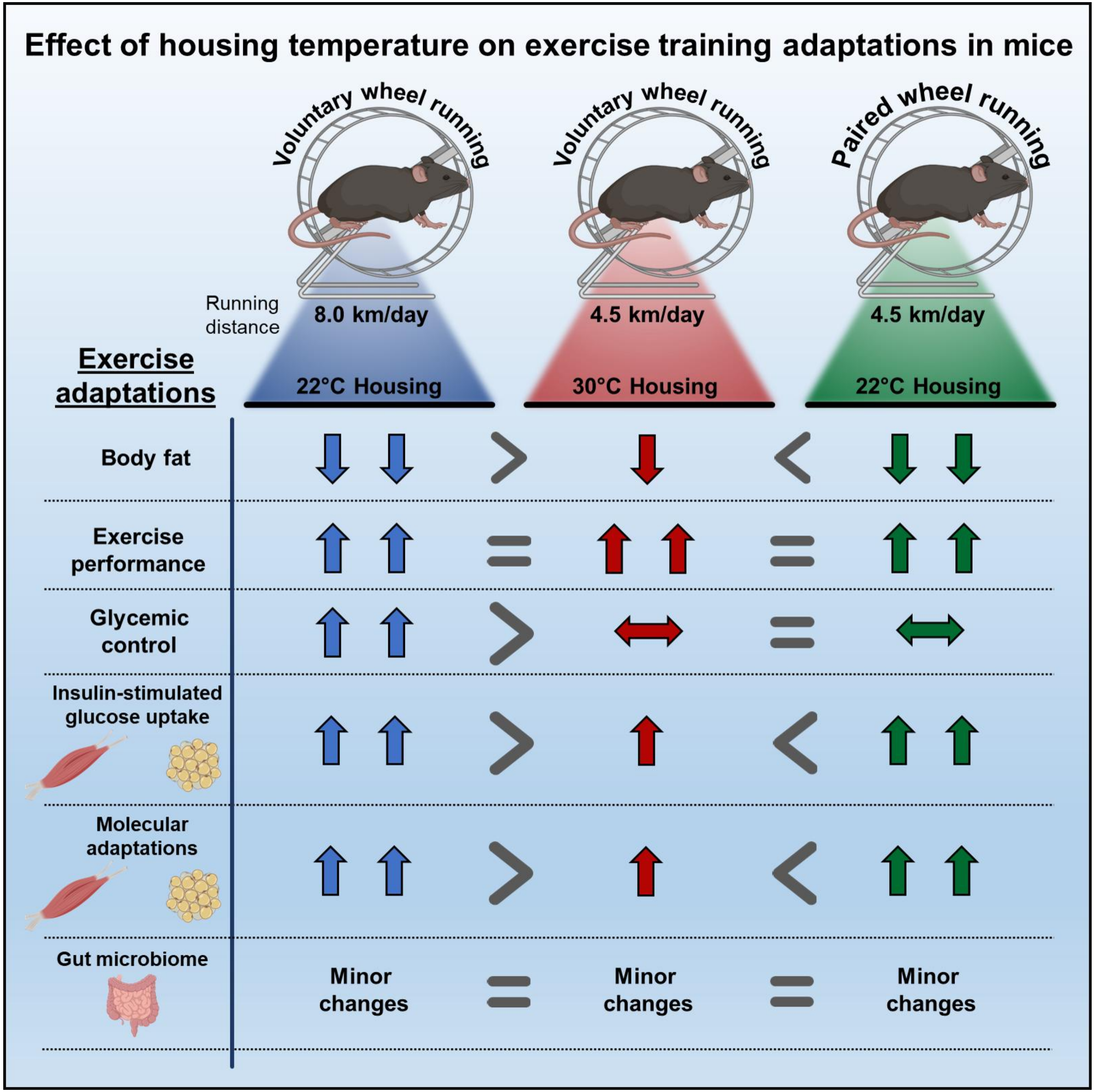

Effect of housing temperature on exercise training adaptations in mice
Voluntary wheel running
Voluntary wheel running
Paired wheel running
Running distance
8.0 km/day
4.5 km/day
4.5 km/day
22°C Housing
30°C Housing
22°C Housing
Exercise adaptations
Body fat
Exercise performance
Glycemic control
Insulin-stimulated glucose uptake
Molecular adaptations
Gut microbiome
Minor changes
Minor changes
Minor changes

Supplementary figure 6:

Graphical abstract
